# Lasso regularization for left-censored Gaussian outcome and high-dimensional predictors

**DOI:** 10.1186/s12874-018-0609-4

**Published:** 2018-12-04

**Authors:** Perrine Soret, Marta Avalos, Linda Wittkop, Daniel Commenges, Rodolphe Thiébaut

**Affiliations:** 1 Univ. Bordeaux, Inserm, Bordeaux Population Health Research Center, UMR 1219, Bordeaux, F-33000 France; 2 Inria SISTM Team, Talence, F-33405 France; 3 Vaccine Research Institute (VRI), Créteil, F-94000 France; 4 CHU Bordeaux, Department of Public Health, Bordeaux, F-33000 France

**Keywords:** Limit of detection, Buckley-James least squares procedure, HIV viral load, Drug resistance, HIV genotypic mutations, Cross-sectional studies

## Abstract

**Background:**

Biological assays for the quantification of markers may suffer from a lack of sensitivity and thus from an analytical detection limit. This is the case of human immunodeficiency virus (HIV) viral load. Below this threshold the exact value is unknown and values are consequently left-censored. Statistical methods have been proposed to deal with left-censoring but few are adapted in the context of high-dimensional data.

**Methods:**

We propose to reverse the Buckley-James least squares algorithm to handle left-censored data enhanced with a Lasso regularization to accommodate high-dimensional predictors. We present a Lasso-regularized Buckley-James least squares method with both non-parametric imputation using Kaplan-Meier and parametric imputation based on the Gaussian distribution, which is typically assumed for HIV viral load data after logarithmic transformation. Cross-validation for parameter-tuning is based on an appropriate loss function that takes into account the different contributions of censored and uncensored observations. We specify how these techniques can be easily implemented using available R packages. The Lasso-regularized Buckley-James least square method was compared to simple imputation strategies to predict the response to antiretroviral therapy measured by HIV viral load according to the HIV genotypic mutations. We used a dataset composed of several clinical trials and cohorts from the Forum for Collaborative HIV Research (HIV Med. 2008;7:27-40). The proposed methods were also assessed on simulated data mimicking the observed data.

**Results:**

Approaches accounting for left-censoring outperformed simple imputation methods in a high-dimensional setting. The Gaussian Buckley-James method with cross-validation based on the appropriate loss function showed the lowest prediction error on simulated data and, using real data, the most valid results according to the current literature on HIV mutations.

**Conclusions:**

The proposed approach deals with high-dimensional predictors and left-censored outcomes and has shown its interest for predicting HIV viral load according to HIV mutations.

## Background

Left-censoring due to the lower detection limit of an assay is a common problem in many fields including biology, chemistry, and the environmental sciences. One example is the quantification of the human immunodeficiency virus (HIV) viral load in plasma. The sensitivity of assays has improved and the detection threshold has decreased from 10,000 copies/mL to 20 or fewer copies/mL today. Several statistical methods have been proposed to account for left-censoring of such quantitative variables in cross-sectional (with one measure per subject) and longitudinal (with several measures per subject) studies. Standard methods include multiple imputation [1–4], reverse survival analysis methods [2, 5–7], quantile regression [8, 9] and censored quantile regression [10, 11]. Furthermore, the Tobit model with censored outcome which is supposed to be normally distributed can be estimated by maximum likelihood [12–18] or by the Buckley-James estimator [18, 19]. Indeed, HIV viral load appears to have an underlying Gaussian distribution truncated by the detection limit that justifies the normality hypothesis [13–15, 17, 18]. As expected, approaches accounting for left-censoring outperform simple imputation of a constant [2, 4, 13–16, 18, 20–22].

Another issue may arise when the number of predictors (*p*) is high compared to the number of statistical units (*n*), without excluding the possibility that *n*<*p*. This is known as high dimensionality. In the context of HIV infection, this can be illustrated by analyzing the association between the presence of HIV mutations and the response to antiretroviral therapy which is measured by HIV viral load. HIV strains circulating in a given individual can present mutations associated with antiretroviral treatment failure (detectable HIV viral load), also called HIV drug resistance mutations. Thus, genotypic tests allowing the detection of HIV drug resistance mutations are commonly performed in patients starting a new antiretroviral regimen or even in newly HIV-infected patients because of the transmission of resistant strains [23–26]. Lasso linear [27, 28] and logistic regressions [29], principal component and partial least square logistic regressions [30], and multiple testing correction [31] have been used to deal with more than 100 predictors and fewer than a few hundred of patients, a common situation in this context [32].

These studies use a dichotomized outcome or simple imputation by a constant to circumvent the problem of censoring. One limitation of dichotomizing a continuous outcome is the loss of information and hence power. In addition, success is usually defined as achieving an undetectable HIV viral load. However, the detection limit, although not random, depends on several factors that differ from one study to another. Thus, there is no reason, except convenience, for the detection limit to correspond to the threshold for dichotomization.

We hypothesize that approaches accounting for left-censoring will exhibit better results compared to simple imputation strategies in a high-dimensional setting similar to what has been found in low-dimensional settings.

Some works have simultaneously addressed both censoring and high-dimensional problems using the Lasso [33–43], partial least squares [44], random forests [45], support vector machines [46], and deep learning [47]. These examples were developed for right-censored survival data. A main approach to left-censored data analysis is based on methods typically used with right-censored survival data such as the Buckley-James estimator. Left-censored data are then previously reversed to right-censored data. While from a statistical point of view, the nature of the outcome (time-to-event or quantitative measurement below a limit of detection) is secondary, this can impact the choice of adequate probability distribution functions and other practical issues.

We propose a Lasso-regularized Buckley-James least squares method with both, non-parametric imputation using Kaplan-Meier and parametric imputation based on the Gaussian distribution. The non-parametric Buckley–James estimator, which simply replaces censored residuals by their conditional expectations in an iterative way, has been previously applied to left-censored HIV viral load data in a cross-sectional study [18]. On the other hand, the Lasso extension of the non-parametric Buckley–James method has been proposed for right-censored data [36, 38, 40, 48]. Our contribution consists in using the latter method for left-censored outcomes and high-dimensional predictors. Furthermore, we propose an original parametric version of the Buckley-James method, which is adapted to the typical assumption of a Gaussian distribution of HIV viral load. We demonstrate the value of these approaches by comparing them to Lasso linear regression with simple imputation [28] for predicting the response to antiretroviral therapy by HIV genotypic mutations.

Our primary objective is to predict as accurately as possible responses in future patients who will switch to a similar regimen. Thus, comparisons are based on mean square prediction error. The prediction performances of the different methods were assessed on simulated data that reproduced the observed data. Then, methods were applied to data obtained in a collaborative study from clinical trials and cohorts provided by the Standardization in Clinical Relevance of HIV Drug Resistance Testing Project from the Forum for Collaborative HIV Research [49]. The actual data presented a moderate censoring rate of 26 %, i.e. a realistic magnitude [18, 50]. However, high (around 50 %) or even severe (around 70 %) censoring rates could be observed in older studies with a high limit of detection (LOD) or particular populations with low treatment failure rate, e.g. HIV controllers [18, 51, 52]. Thus, we also explored the impact of high and severe censoring rates on performance.

We detail how to use publicly available R packages to compute Lasso estimates with left-censored data.

Finally, we discuss possible extensions and applications of our work.

## Methods

### Methods to analyze left-censored outcome

In this section, we review the simplest models and estimation methods used to deal with left-censoring in cross-sectional studies. For a more extensive and comprehensive review of these methods, see [2, 18]. Thereafter, we consider the Lasso extension of those methods that support simple implementations.

#### The linear model

First, consider the general linear regression model

1 $$ Y_{i} = \mathbf{X}_{i} \boldsymbol{\beta} + \varepsilon_{i}, \hspace{0.5cm} i = {1, \cdots, n}   $$

where **X**_*i*_ is a *p*-vector of fixed predictors, *Y*_*i*_ is the uncensored continuous random outcome variable, ***β***=(*β*_1_,⋯,*β*_*p*_)^⊤^ is a *p*-vector of unknown regression parameters and *ε*_*i*_ are independent and identically normally distributed random variables with mean 0 and constant variance *σ*^2^. Let **X** be the *n*×*p* matrix **X**=(**X**_1_,…,**X**_*n*_)^⊤^ and **Y** the *n*×1 vector **Y**=(*Y*_1_,…,*Y*_*n*_)^⊤^. The intercept is omitted in the model for simplicity, and all predictor variables are assumed to be standardized (i.e. zero mean and unit variance).

#### Lasso on complete data

The Lasso (Least Absolute Shrinkage and Selection Operator) [53] is one of the most popular methods in high-dimensional data analyses. It allows for simultaneous estimation and variable selection and has efficient algorithms available. It is considered here as the *Gold Standard* for our simulation studies. The Lasso estimator of parameters in model (1) is:

2 $$ \hat{\boldsymbol{\beta}}(\lambda)_{GoldS} = \underset{\boldsymbol{\beta}}{\text{argmin}} \left\|\mathbf{Y} - \mathbf{X} \boldsymbol{\beta}\right\|^{2}_{2} + \lambda \left\| \boldsymbol{\beta} \right\|_{1}   $$

where $\left \| \mathbf {Y} - \mathbf {X} \boldsymbol {\beta } \right \|^{2}_{2} = {\sum \nolimits }_{i=1}^{n} \left (Y_{i} - \mathbf {X}_{i} \boldsymbol {\beta } \right)^{2}$ is the quadratic loss, $\left \| \boldsymbol {\beta } \right \|_{1} = {\sum \nolimits }_{j=1}^{p} \left | \beta _{j} \right |$ is the Lasso penalty on the parameter size, and *λ*>0 controls the amount of regularization. When *λ* is large enough (which depends on data), all coefficients are forced to be exactly zero. Inversely, *λ*=0 corresponds to the unpenalized ordinary least-squares estimate.

This model on complete data (no left-censored measures) is considered as a reference when comparing the other methods applied to incomplete datasets that include left-censored values.

#### The Tobit model

Because of the detection limit, *Y*_*i*_ can be left-censored. Let LOD_*i*_ be the (fixed and known) censoring threshold of subject *i*. To simplify, we consider LOD_*i*_=LOD. *Z*_*i*_ is the observed response. The so-called Tobit model [12] can be defined as:

3 $$ Z_{i} = \left\{ \begin{array}{ll} Y_{i} \hspace{1.05cm} \text{if} \hspace{0.5cm} Y_{i} > \text{LOD} \\ \text{LOD} \hspace{0.65cm} \text{if} \hspace{0.5cm} Y_{i}\leq \text{LOD} \end{array} \right.   $$

where *Y*_*i*_ is the response variable defined in model (1). We can equivalently write:

4 $$ \begin{array}{lll} Z_{i} = \max\left(Y_{i}, \text{LOD}\right), & \text{or} & Z_{i} = \delta_{i} Y_{i}+\left(1-\delta_{i}\right)\text{LOD}, \end{array}   $$

where $\delta _{i} = \mathbb {I}_{\left (Y_{i} > \text {LOD}\right)}$ is a censoring indicator. The idea behind the Tobit regression model is to deal with the left-censored variable *Z* as the outcome of a normally distributed latent variable *Y*.

#### Simple imputation

Simple imputation is a substitution method that replaces left-censored values with a single value, LOD. LOD/2 is another common choice. Let $\hat {\boldsymbol {\beta }}_{LOD}$ be the ordinary least squares estimate of model:

5 $$ Z_{i} = \mathbf{X}_{i} \boldsymbol{\beta} + \varepsilon_{i}, \hspace{0.5cm} i = {1, \cdots, n}   $$

Simple imputation is widely used for its simplicity. However, replacing any censored observation by a single value may lead to biased parameter estimates.

Beerenwinkel et al. [28] applied Lasso-regularized linear regression with the naïve approach of replacing the unobserved undetectable value with the limit of detection of the assay. Then the Lasso estimator of parameters in model (5) is:

6 $$ \hat{\boldsymbol{\beta}}(\lambda)_{LOD} = \underset{\boldsymbol{\beta}}{\text{argmin}} \left\|\mathbf{Z} - \mathbf{X} \boldsymbol{\beta}\right\|^{2}_{2} + \lambda\left\|\boldsymbol{\beta}\right\|_{1}.  $$

#### Maximum likelihood estimation

In the Tobit model (1)-(3), one can assume that when *Z*= LOD, the density function of *Z* is equal to the probability of observing *Y*≤ LOD and for *Z*>*LOD* the density function of *Z* is the same as the density of *Y*. The likelihood function takes the form:

$${}\mathcal{L}\left(\boldsymbol{\beta}, \sigma^{2} \right) \,=\, \prod\limits_{i=1}^{n}\! \mathbb{P}\!\left(Y_{i} \left| Y_{i} \!>\!\! \text{LOD}, \mathbf{X}_{i}\right.\right)^{\delta_{i}} \mathbb{P}\!\left(Y_{i} \left| Y_{i} \!\leq\! \text{LOD}, \mathbf{X}_{i}\right.\!\right)^{1 - \delta_{i}}  $$

When the Gaussian distribution for the outcome is assumed, the log-likelihood function can be written as:

7 $$\begin{array}{*{20}l} {}\text{ln}\mathcal{L}\left(\boldsymbol{\beta}, \sigma^{2}\right) & \,=\, \sum\limits_{i=1}^{n} \delta_{i} \ln f_{G}\left(Y_{i},\mathbf{X}_{i},\boldsymbol{\beta}, \sigma^{2}\right) \\ &\quad + \left(1-\delta_{i}\right) \ln F_{G}\left(\text{LOD},\mathbf{X}_{i},\boldsymbol{\beta}, \sigma^{2}\right)  \end{array} $$

with $f_{G}\left (u, \mathbf {X}_{i}, \boldsymbol {\beta }, \sigma ^{2}\right) = \frac {e^{\frac {\left (u - \mathbf {X}_{i} \boldsymbol {\beta }\right)^{2}}{-2 \sigma ^{2}}}}{\sqrt {2 \pi \sigma ^{2}}}$ the Gaussian probability density function of *Y*_*i*_ with mean **X**_*i*_***β*** and constant variance *σ*^2^ evaluated at *u* and $F_{G}\left (v, \mathbf {X}_{i}, \boldsymbol {\beta }, \sigma ^{2}\right) = \int _{- \infty }^{v}f_{G}\left (u, \mathbf {X}_{i}, \boldsymbol {\beta }, \sigma ^{2}\right) \, \mathrm {d}u $ is the corresponding Gaussian cumulative distribution function evaluated at *v*. Let $\hat {\boldsymbol {\beta }}_{MLE}$ be the maximum likelihood estimation obtained by maximizing (7). Extensions to other distributions have also been explored [54]. Several works have shown the superiority of this method [18, 20–22]. However, when the parametric model is misspecified, the sample size is small or the percent censoring is high, the maximum-likelihood estimation method has been shown to perform poorly [2].

The Lasso penalty applied to some likelihood function has become an established and relatively standard technique. However, when the likelihood function is a more complex function of the model parameter, such as the likelihood function for the Tobit model (7), adding a non-differentiable penalty leads to a computational challenging optimization.

#### Quantile regression and censored quantile regression

Quantile regression, particularly least absolute deviations (LAD) regression, has been applied to left-censored data [9]:

$$\hat{\boldsymbol{\beta}}_{LAD} = \underset{\boldsymbol{\beta}}{\text{argmin}}\,\,\, \frac{1}{n} \sum\limits_{i=1}^{n} \left| Z_{i} - \mathbf{X}_{i} \boldsymbol{\beta} \right|  $$

Median regression is a natural alternative to the usual mean regression in the presence of heteroscedasticity or when the normality assumption is violated. Simple imputation using robust regression may be less sensitive to the influence of censored observations.

Lasso-regularized least absolute deviations regression has been investigated in the literature (e.g. [55]).

Powell [10] proposed the LAD estimate specifically for censored data:

$$\hat{\boldsymbol{\beta}}_{CLAD} = \underset{\boldsymbol{\beta}}{\text{argmin}}\,\,\, \frac{1}{n} \sum\limits_{i=1}^{n} \left| Z_{i} - \max\left\{ \text{LOD}, \mathbf{X}_{i} \boldsymbol{\beta} \right\} \right|  $$

Later, the approach was extended to more general quantiles [11].

The Lasso extension of censored quantile regression (basically, censored LAD) has been analyzed for right-censored survival data [41, 42, 56] and specifically for left-censored data [57–62]. Yu et al. [58] and Alhamzawi et al. [61] proposed Bayesian approaches using different hyperparameters priors. These methods rely on computationally intensive algorithms. In practice, applications are limited to the *n*>>*p* case. Others [57, 59, 60, 62] derived theoretical properties of the Lasso-regularized censored least absolute deviations regression, but the algorithmic development was not a priority in these works and the practical use was limited to *n*>>*p* or not addressed. To our knowledge, there are no publicly available software tools that implement the Lasso extension of Powell’s approach and no simple implementation relying on existing packages seems straightforward.

#### Non-parametric Buckley-James

Left-censored outcome data can be analyzed using methods designed for right-censored survival data by reversing the outcome scale. For instance, Gillespie et al. [6] proposed the reverse Kaplan-Meier and Dinse et al. [7] reversed the Cox method (though in the case of left-censored exposures and uncensored outcome). After the Cox model, the accelerated failure time model is the most frequently used regression model for right-censored survival data. It directly links the expected response to predictors, analogously to the classical linear regression approach. A popular method for fitting the accelerated failure time model is the Buckley-James estimator [19], an extension of the least squares principle. The idea is to impute the censored values by their estimated conditional mean to provide censoring and predictor values:

8 $$ Z_{i}^{*} = \delta_{i} Y_{i}+\left(1-\delta_{i}\right)\mathbb{E} \,\left(Y_{i} \left| Y_{i} \leq \text{LOD}, \mathbf{X}_{i}\right.\right)   $$

with

$$\mathbb{E} \,\left(Y_{i} \left| Y_{i} \leq \text{LOD}, \mathbf{X}_{i}\right.\right) = {\int\nolimits}_{-\infty}^{\text{LOD}} \frac{uf\left(u, \mathbf{X}_{i}, \boldsymbol{\beta}\right)\mathrm du}{F\left(\text{LOD}, \mathbf{X}_{i}, \boldsymbol{\beta}\right)}  $$

where *f*(*u*,**X**_*i*_,***β***) is the (unknown) probability density function of *Y*_*i*_ with mean **X**_*i*_***β*** evaluated at *u* and *F*(*u*,**X**_*i*_,***β***) is the corresponding cumulative distribution function. By "flipping" the data (turning it from left-censored to right-censored), the application of algorithms previously developed for right-censoring is direct and has been performed in other contexts [5]. We consider *M* an arbitrary constant that equals or exceeds the largest observation. Then subtract all uncensored and left-censored outcomes from *M*. The left-censored at LOD variable **Z** is then replaced by *M*−**Z** which is right-censored at *M*−LOD. Let (*M*−*Z*_*i*_)^∗^ be imputed as $\delta _{i} \left (M-{Y}_{i}\right) + \left (1 - \delta _{i}\right)\, \mathbb {E} \,\left (M-Y_{i} \left | M-{Y}_{i}\geq M-\text {LOD}, \mathbf {X}_{i}\right.\right)$. Then, we can calculate the conditional expectation by

9 $$ \begin{aligned} \mathbb{E} &\left(M-Y_{i} \left| M-Y_{i} \geq M-\text{LOD}, \mathbf{X}_{i}\right.\right) \\&= {\int\nolimits}_{M-\text{LOD}}^{\infty} \frac{uf\left(u, \mathbf{X}_{i}, \boldsymbol{\beta}\right)\mathrm du}{1-F\left(M-\text{LOD}, \mathbf{X}_{i}, \boldsymbol{\beta}\right)}  \end{aligned}  $$

where *F*(*u*,**X**_*i*_,***β***) is now the (unknown) cumulative distribution function of *M*−*Y*_*i*_ with mean *M*−**X**_*i*_***β*** evaluated at *u*, which can be estimated, for example, by Kaplan-Meier. The Buckley-James estimate, $\hat {\boldsymbol {\beta }}_{NonParBJ}$, can be computed using a semiparametric iterative algorithm that alternates between imputation of censored values according to (9) and least-squares estimation.

The main drawback of this method is that convergence of the algorithm is not guaranteed. Due to the discontinuous nature of the estimating function (formulation (8) makes $\hat {\boldsymbol {\beta }}_{NonParBJ}$ to be a piecewise linear function in ***β***), the iterative procedure may oscillate between different parameter values. The problem is of practical importance in situations where the effect of predictors is small or in small samples [63] (which could be worse in high-dimensional settings). To circumvent this problem, a one-step algorithm that stops at the first iteration is used in some works [36, 64]. This approach is close to a substitution method in which values below the detection limit are replaced by expected values of the missing measurements, provided they are less than the detection limit [65].

Several authors have proposed combining the iterative Buckley-James imputation and methods handling high-dimensional predictors: Johnson et al. [36, 48] and Cai et al. [38] used the Lasso, Wang et al. [37], used boosting, Wang et al. [40] used ElasticNet, Johnson et al. [66] and Li et al. [67] used the Dantzig selector, and Dirienzo et al. [68] used parsimonious covariate selection. The Buckley-James estimate can be computed using an iterative algorithm that alternates between imputation of censored values according to (9) and the Lasso:

10 $$ {}\hat{\boldsymbol{\beta}}(\lambda)_{NonParBJ} \,=\, \underset{\boldsymbol{\beta}}{\text{argmin}} \left\| (M\,-\,\mathbf{Z})^{*} \,-\, (M\,-\,\mathbf{X} \boldsymbol{\beta}) \right\|_{2}^{2} + \lambda \left\| \boldsymbol{\beta} \right\|_{1}   $$

#### Gaussian Buckley-James

Alternatively, the Buckley-James imputation (8), assuming the logarithm of HIV viral load follows a Gaussian distribution, can be calculated with the conditional expectation:

11 $$ \mathbb{E} \,\left(Y_{i} \left| Y_{i} \leq \text{LOD}, \mathbf{X}_{i}\right.\right) = {\int}_{-\infty}^{\text{LOD}} \frac{{uf}_{G}\left(u, \mathbf{X}_{i}, \boldsymbol{\beta}, \sigma^{2}\right)\mathrm{d}u}{F_{G}\left(\text{LOD}, \mathbf{X}_{i}, \boldsymbol{\beta}, \sigma^{2}\right)}   $$

where *f*_*G*_ and *F*_*G*_ are the Gaussian density and cumulative distribution functions defined in (7). Again, the solution can be computed by iteratively alternating between imputation based on (11) and parameter estimation using ordinary least squares, $\hat {\boldsymbol {\beta }}_{GaussBJ}$, or the Lasso:

12 $$ \hat{\boldsymbol{\beta}}(\lambda)_{{GaussBJ}} = \underset{\boldsymbol{\beta}}{\text{argmin}} \left\| \mathbf{Z}^{*} - \mathbf{X} \boldsymbol{\beta} \right\|_{2}^{2} + \lambda \left\| \boldsymbol{\beta} \right\|_{1}   $$

#### Graphical illustration in a low-dimensional setting

To illustrate the difference between estimation methods we generated data from the simple linear model (*p*=1): *Y*_*i*_=*X*_*i*_*β*+*ε*_*i*_ with *i*=1,⋯,*n*, **X**∼*N*(0,1), ***ε***∼*N*(0,*σ*^2^). *β* was set to 10 and *σ*^2^ was chosen such that the signal-to-noise ratio was 4 :3. A limit of detection was then fixed to obtain the desired censoring rate: moderate, 20 %, high, 50 % or severe, 70 %.

**Fig. 1:**
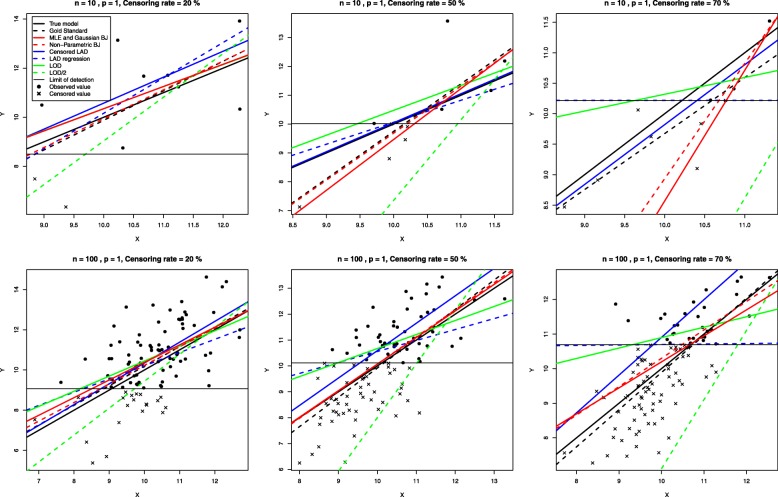
Observed and predicted simulated censored data. Regression lines are obtained using: the true model that generated the data, the gold standard, maximum likelihood estimation (MLE), non-parametric Buckley-James (BJ), least absolute deviations (LAD) and censored LAD regressions, simple imputation by the limit of detection (LOD) and by LOD /2

In Fig. 1, predicted regression lines are obtained using different methods: the true model that generated the data, the gold standard (ordinary least squares with uncensored data), maximum likelihood estimation (MLE), which is identical to the Gaussian Buckley-James estimation (BJ) when *p*=1, non-parametric Buckley-James (BJ), least absolute deviations (LAD) and censored LAD regressions, simple imputation by the limit of detection (LOD) and by LOD /2.

Notice that simple imputation by LOD and LOD/2 are the most distant regression lines from the true and gold standard lines, in an opposite way: simple imputation by LOD tends to overestimate the response values while simple imputation by LOD/2 tends to underestimate them.

Maximum likelihood estimation shows one of the best behaviors, but the computational complexity dramatically increases with *p* (results not showed). Mean and median regressions with simple imputation by LOD are quite close and are the closest when the estimation situation is easy (high *n*, low censoring rate). The censored LAD shows better results than censored mean regression (MLE and Buckley-James) for small sample size while the inverse is observed when *n*=100. Gaussian Buckley-James and MLE are identical, but their differences increase when *p* increases (results not showed). In this i.i.d. generated from a Gaussian distribution example, the Gaussian Buckley-James estimate shows better behavior than non-parametric Buckley-James, the difference being higher when *n* is small.

### Tuning parameter selection

K-fold cross-validation is routinely applied to select the optimal regularization parameter when the main goal of the study is prediction. Data **D** is randomly chunked into *K* disjoint blocks of approximately equal size. To avoid a potentially unbalanced partition, we consider stratified K-fold cross-validation, i.e. each fold contains roughly the same proportion of censoring as in the whole sample. $\mathbf {D}_{\smallsetminus k}$ is the learning data, used to estimate coefficients. **D**_*k*_ is the test data, not used in the estimation process and then used to evaluate the loss function *L*. This K-fold cross-validation can be written as:

13 $$ \text{CV}({\lambda}) = \frac{1}{K} \sum\limits_{k=1}^{K} {L}\left(\hat{\boldsymbol{\beta}}(\lambda)_{\mathbf{D}_{\smallsetminus k}}, \mathbf{D}_{k}\right)  $$

CV is evaluated on a grid of *λ*–values. The highest value, *λ*_max_, corresponds to the smallest value of *λ* for which all coefficients are zero. The lowest value, *λ*_min_, corresponds to the unpenalized solution (when feasible). We choose the *λ* value that minimizes the CV function.

Squared error loss is one of the most widely used loss functions:

14 $$ {L}(\hat{\boldsymbol{\beta}}(\lambda)_{\mathbf{D}_{\smallsetminus k}}, \mathbf{D}_{k})= \frac{1}{n_{k}} \sum_{i\in \mathbf{D}_{k}} \left(Y_{i} - \mathbf{X}_{i} \hat{\boldsymbol{\beta}}(\lambda)_{\mathbf{D}_{\smallsetminus k}} \right)^{2},   $$

where *n*_*k*_ is the sample size of **D**_*k*_. However, **Y** is a latent variable not fully observed due to the detection limit. This loss function could be used only for the gold standard in (2), with simulated data. Again, the simplest imputation strategy consists in replacing **Y** with **Z**, in **D**_*k*_. Alternatively, Buckley-James strategies could replace censored *Y*_*i*_ values in the test data **D**_*k*_ by their conditional expectation estimated using the learning data [48].

On the other hand, a loss function differentiating the contribution of uncensored and censored data would be useful. Assuming the Gaussian distribution of the HIV viral load (7), the following loss function could be derived:

15 $$ \begin{array}{ll} &{}{ {L}_{G}\left(\hat{\boldsymbol{\beta}}(\lambda)_{\mathbf{D}_{\smallsetminus k}}, \mathbf{D}_{k}\right)=} {\frac{1}{n_{k}^{\text{unc}}}\sum\limits_{{i\in\mathbf{D}_{k}} \atop i\text{ uncensored}}\left(Y_{i} - \mathbf{X}_{i} \hat{\boldsymbol{\beta}}(\lambda)_{\mathbf{D}_{\smallsetminus k}} \right)^{2} +} \\\\ &{\frac{2\hat\sigma^{2}_{\mathbf{D}_{\smallsetminus k}}}{n_{k}^{\text{unc}}} \sum\limits_{{i\in\mathbf{D}_{k}} \atop i\text{ censored}}-\ln F_{G}\left(\text{LOD},\mathbf{X}_{i},\hat{\boldsymbol{\beta}}(\lambda)_{\mathbf{D}_{\smallsetminus k}},\hat\sigma^{2}_{\mathbf{D}_{\smallsetminus k}}\right)} \end{array}   $$

where $n_{k}^{\text {unc}}$ is the number of uncensored observations in **D**_*k*_. The loss function *L*_*G*_ in (15) is proportional and equivalent to the negative Gaussian log-likelihood loss function, but allows for comparison with the squared loss in (14).

### Implementation issues

All statistical analyses, comparisons and implementations were performed using the computing environment R (R Development Core Team, 2017) [69]. We used the function cv.glmnet from package glmnet [70] to choose the optimal *λ* value of Lasso linear regression on complete data (*GoldS*) and Lasso linear regression with simple substitution of left-censored values by the detection limit (*LOD*). We implemented the Lasso non-parametric Buckley-James (*NonParBJ*) using the bujar package [71]. We modified the function to support stratified K-fold cross-validation and conserve the same proportion of censoring in all the folds. Lasso Gaussian Buckley-James (*Gaussian BJ*) was implemented in a new function cvGaussBJ. Algorithm 1 specifies how to solve the problem. The stopping criterion is based on the difference between current and previous regression coefficient estimates, variance estimates, and imputed data. Because of the tendency to oscillate between different parameter values of the iterative procedure, the algorithm is also stopped if the number of oscillations is high [40]. Alternatively, we also considered the one-step algorithm, which stops at the first iteration [36, 64]. Cross-validation based on both imputation and loss function accounting for censored and uncensored contributions is considered. The Lasso estimation step depends on package glmnet.

All these implementations and an artificial example are available at: https://github.com/psBiostat/left-censored-Lasso.

### Prediction of HIV viral load from HIV genotypic mutations: real and simulated data

HIV is highly replicative and thus presents high mutation and recombination rates which could lead to the development of HIV drug resistance and consequently reduce the efficacy of antiretroviral treatment. To optimize the control of the evolution of HIV drug resistance, HIV viral load is routinely monitored to identify treatment failure, and HIV genotypic tests are commonly performed before a switch to a new treatment regimen in patients already treated or at the initiation of the first treatment in naive HIV-infected patients [72].


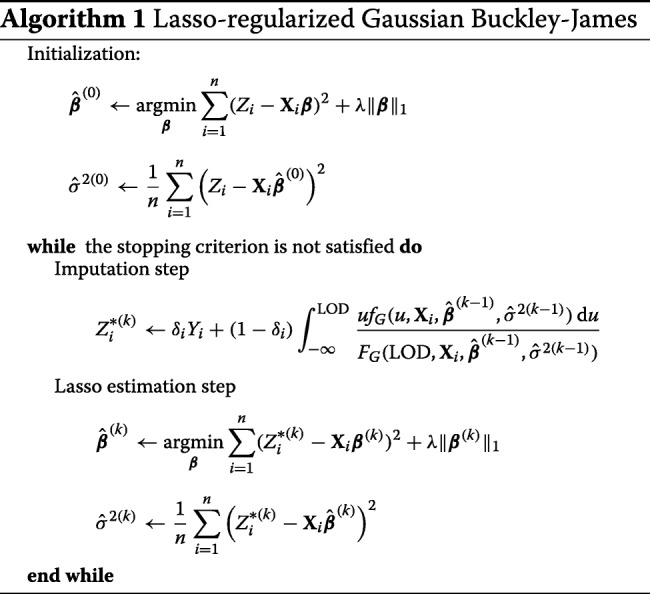


Our objective is to compare methods that handle left-censoring by conditional imputation with methods that handle left-censoring by imputing a single constant value (that is, the Lasso-regularized linear regression with simple imputation by LOD and LOD /2) to predict of HIV viral load by HIV genotypic mutations. The methods accounting for left-censoring by imputing the estimated conditional mean given censoring and predictor values are the Lasso-regularized Buckley-James least square algorithms (with/without Gaussian assumption, with complete convergence/1-step, using cross-validation based on imputation/loss function accounting for censored and uncensored contributions).

#### Real data

The database used in this study was provided by the Standardization and Clinical Relevance of HIV Drug Resistance Testing Project for the Forum for Collaborative HIV Research [49]. Patients included in this study were all treatment-experienced and switched to an abacavir-containing regimen. The investigated drug, abacavir, is a nucleoside reverse transcriptase inhibitor (NRTI) that blocks HIV reverse transcriptase.

The sample size *n*=99 was slightly smaller than the number of predictors *p*=121. 54 of the 121 predictors correspond to the presence or absence of specific mutations in the reverse transcriptase gene (RTG), which were reported to be probably associated with resistance to abacavir, multi-NRTI, NRTI (other than abacavir) or non-nucleoside reverse transcriptase inhibitors (NNRTI) at the time of the study [73, 74]. The number of mutations reported to be probably associated with resistance to abacavir or multi-NRTI is low (14%). The other 67 predictors correspond to the presence or absence of specific mutations in the protease gene (PG) reported to be probably associated with resistance to one or several protease inhibitors (PI) at the time of the study [73, 74]. The number of molecules, including abacavir, ranged from 1 to 6 (with the median number of molecules being 3 and interquartile range 2). In particular, a PI was prescribed in 59% of the patients and 43% received an NNRTI. The response variable is the log-HIV viral load measured at *t*_8_ (8 weeks after treatment initiation at *t*_0_). LOD was fixed at 100 copies/mL and the censoring rate was moderate (26%).

#### Generation of simulated data

HIV viral load appears to have an underlying Gaussian distribution when log-transformed. Therefore, our outcome, $Y^{(8)}_{i}$ is generated from a Gaussian distribution. We simulated 200 data sets of size *n*=100 and *p*=100 predictors from the model:

$$Y^{(8)}_{i} = \beta_{0} + \beta_{1}^{(0)} Y_{i}^{(0)} + \mathbf{X}_{i} \boldsymbol{\beta} + \varepsilon_{i} \,\,\,\,\,\,\,\,\,\, i=1, \cdots, n $$

where

- $Y_{i}^{(0)}$, the HIV viral load at *t*_0_ is generated by a normal distribution with mean 12 (log10 copies/mL) and variance 1.
- $\beta _{1}^{(0)}$ represents the change of the slope between the HIV viral load on the day of treatment, *t*_0_, and 8 weeks later, at *t*_8_, when no mutations are present and for 1 *log*_10_/mL higher concentration of viral load at *t*_0_.
  We fix *β*_0_ and $\beta _{1}^{(0)}$ to obtain the desired censoring rates: 20% (moderate), 50% (high), and 70% (severe).
- **X**_(*n*×*p*)_, representing the presence or absence of HIV mutations, is generated by a multinomial distribution with mean 0.15 (the fixed prevalence for all the 100 mutations) and covariance matrix ***Σ*** where *Σ*_*ij*_=0.4^|*i*−*j*|^ (the closer the mutations, the more positively they are correlated).
- ***β***=(*β*_1_,⋯,*β*_*p*_)^⊤^. Among *p*=100 candidate mutations only 10% are relevant with effects *β*_*j*_=1, if *j*=1,…,10 and 0 if *j*>10. A 1-unit increase in HIV viral load is expected per occurrence of these relevant mutations for a given baseline HIV viral load.
- ***ε*** is generated from a normal distribution with mean 0 and variance *σ*^2^ chosen such that the signal-to-noise ratio is fixed at 3:1.

Our primary goal was to compare competing methods in terms of prediction accuracy. Consequently, we simulated training and test datasets. The former were used to estimate, the latter were used to evaluate the prediction performance. We ensured that training and test datasets contained roughly the same proportions of censoring. We computed the mean squared error on test data as:

$$\text{MSE} = \frac{1}{n^{\text{test}}} \sum\limits_{i=1}^{n^{\text{test}}} \left(Y_{i}^{\text{test}} - \mathbf{X}_{i}^{\text{test}}\hat{\boldsymbol{\beta}}(\hat\lambda)\right)^{2} $$

with $\hat {\boldsymbol {\beta }}(\hat \lambda)$ estimated on training data by stratified 5-fold cross-validation using a given regression method (Gold standard, Lasso-regularized Non-parametric and Gaussian Buckley-James -with complete convergence and 1-step- and Lasso-regularized linear regression with simple imputation -by LOD and LOD /2-) and the corresponding loss function in the cross-validation criterion. The gold standard uses (14), the others replace the censoring values according to their imputation strategy, and the Gaussian Buckley-James also uses the loss function in (15).

## Results

### Simulation results

**Fig. 2:**
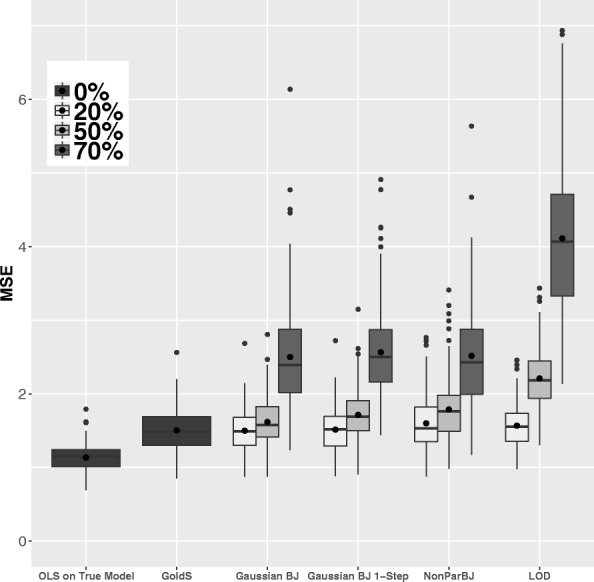
Mean Square Error (MSE) calculated on test data of size *n*_*test*_=100 and 200 replications using gold standard method (*GoldS*), Gaussian Buckley-James (*Gaussian BJ*) and 1-Step version (*Gaussian BJ 1-Step*), non-Parametric Buckley-James (*NonParBJ*) and simple imputation by LOD (*LOD*). Data were generated to present different censoring rates: 0% (uncensored), 20% (moderate), 50% (high), and 70% (severe), which are represented by different gray degrees

Figure 2 shows the mean prediction error results. "OLS on True Model" corresponds to ordinary least squares for the linear model with uncensored data and only relevant predictors (the so-called oracle estimator). Gold standard (*GoldS*) corresponds to the Lasso estimation for the linear model with uncensored data. These results allow for reference prediction errors when the true model is known (the first one) or due to censoring data (both).

The imputation by LOD /2 led to poorer results than the imputation by LOD. Thus, for the simple imputation, only LOD imputation (*LOD*) results are shown. For the Gaussian Buckley-James algorithms (*Gaussian BJ*), the error is calculated by using both cross-validation with imputation and cross-validation with the loss function indicated in (15), but only the best results are shown.

The Gaussian Buckley-James method presented an oscillating behavior in 9.5*%* of the generated samples when the convergence rate was 20%. This percentage rose to 82.5*%* and 95.0*%* when the convergence rates were 50% and 70%, respectively. For the Gaussian Buckley-James using the 1-step algorithm (*Gaussian BJ 1-Step*), results using the two cross-validation approaches were almost identical. Nevertheless, for the Gaussian Buckley-James with complete convergence, a notable improvement was obtained when applying (15).

The higher the rate of censoring, the less information is available to train the models and, unsurprisingly, the higher is the prediction error. For a moderate rate of censoring (20%), all methods show a good performance close to that of the gold standard *GoldS*. When the rate of censoring is 50%, *Gaussian BJ* shows the lowest prediction error, followed by *Gaussian BJ 1-step*, *NonParBJ* and finally simple imputation, which shows more errors. The same patterns but more pronounced were observed with a severe rate of censoring (70%). Taking the knowledge about the distribution into account appears to have only a slight impact. Simple imputation yields the poorest results. In addition, it showed high variability with some extreme errors.

### Application to real data

The Lasso-regularized Buckley-James least square algorithm that showed the best behavior in the simulation study (with complete convergence and cross-validation based on the loss function *L*_*G*_ in (15)) was applied to real data, as well as the Lasso-regularized non-parametric Buckley-James method and simple imputation (by LOD and LOD /2).

Regularization parameters were estimated by stratified cross-validation in order to ensure that each fold had the same proportion of censoring as in the corresponding data set (26%). In addition, because some mutations were relatively infrequent, we used 20-fold cross-validation. Indeed, the higher the number of folds, the lower the probability of randomly obtaining test sets with no subject exposed to infrequent HIV drug resistance mutations.

**Fig. 3:**
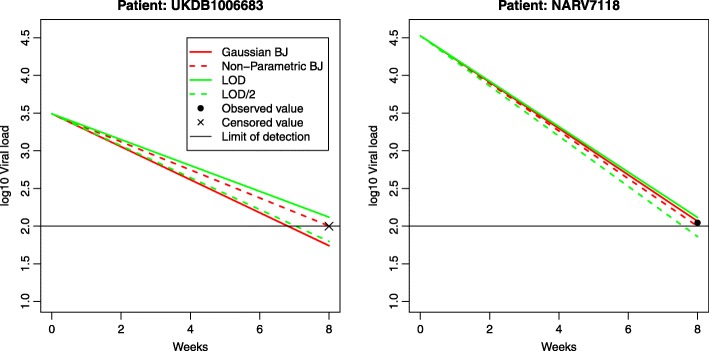
Two examples of individual predicted values using Lasso regularization techniques (Gaussian Buckley-James, non-parametric Buckley-James, simple imputation by LOD and LOD/2) and observed values. One example is censored (at left) and the other is uncensored (at right)

Figure 3 shows two examples of the observed HIV viral load at *t*_0_ and the observed and estimated HIV viral load at *t*_8_. As in the low-dimensional case shown in Fig. 1, simple imputation by LOD predicted the highest values of HIV viral load. Inversely, simple imputation by LOD /2 estimates the lowest values of HIV viral load. The difference between the two estimates at 8 weeks is >0.5 *log*_10_ copies/mL, which is clinically relevant. Lasso-regularized Buckley-James least square algorithms (with/without Gaussian assumption), often gave a prediction in between. This tendency was increased when the censoring rate was high or when the sensitivity of the assay was low.

When applying the Gaussian Buckley-James method to real data, no oscillating behavior was observed.

Table 1 indicates the number of HIV genotypic mutations selected from the list of mutations that may contribute to a reduced virologic response known at the time of the study [73, 74], according to each method applied. The Lasso-regularized Gaussian Buckley-James selected several HIV genotypic mutations suspected of being associated with abacavir or multi-NRTI resistance. Furthermore, it selected a high number of HIV genotypic mutations probably associated with PI resistance and a few probably associated with NNRTI resistance. This selection of a large number of candidate predictors seems to be relevant because all patients received an abacavir-containing regimen and a high percentage of patients received regimens including a PI- and/or NNRTI. The Lasso-regularized non-parametric Buckley-James selected fewer mutations, and especially fewer mutations in PG, probably due to PI resistance. Simple imputation of the LOD or LOD /2 selected few mutations. In particular, only 1 of the 5 mutations in RTG probably associated with abacavir resistance was retained.

**Table 1:** Distribution of 121 HIV genotypic mutations included in real data study according to knowledge at study time and reported in [73, 74] and number of HIV genotypic mutations selected by Lasso regularized methods

Number of HIV genotypic mutations	Gaussian BJ	NonPar BJ	LOD	LOD/2
present in real data study				
5 in RTG probably associated	3 (60%)	3 (60%)	1 (20%)	1 (20%)
with abacavir resistance				
13 in RTG probably associated	7 (54%)	4 (31%)	4 (31%)	4 (31%)
with multi-NRTI resistance				
6 in RTG probably associated	3 (50%)	2 (33%)	1 (17%)	1 (17%)
with NRTI resistance (other than abacavir)				
30 in RTG probably associated	12 (40%)	11 (37%)	8 (27%)	7 (23%)
with NNRTI resistance				
67 in PG probably associated	40 (60%)	22 (33%)	15 (22%)	14 (21%)
with PI resistance				
121 Total	65 (54%)	42 (35%)	29 (24%)	27 (22%)

## Discussion

Simple imputation of the detection limit or of half of this limit is an ad hoc approach to address left-censored outcome data. However, in standard (low-dimensional) settings, it leads to biased estimates of parameters and standard errors. In our high-dimensional simulation study, simple imputation using Lasso-regularized least-squares showed poor performance. As in low-dimensional settings, approaches accounting for left-censoring outperformed simple imputation.

In this work, we propose a Lasso-regularized Gaussian Buckley-James algorithm, according to the usual Gaussian assumption of log-transformed HIV viral load. Because of the well-known convergence problems of the iterative Buckley-James procedure, we implemented two algorithms, the first algorithm running until convergence and the second one being stopped after one step [36, 64]. This one-step algorithm showed similar results in the simulation study. Other solutions have been proposed to deal with convergence problems in low-dimensional settings [39, 64] and could be investigated in future research.

As in other works [48], we implemented a cross-validation criterion for the tuning parameter based on imputing values to *Y*_*i*_ in the test set from conditional expectations estimated using the learning set. We also proposed a cross-validation criterion based on a loss function that accounts for the different contribution of censored and uncensored values. Almost identical results were obtained when applying the two cross-validation criteria to the one-step algorithm. However, when running the algorithm until convergence, better results were obtained with the cross-validation criterion based on a loss function that accounts for censored and uncensored contributions.

On the other hand, we reversed the Lasso-regularized non-parametric Buckley-James method previously applied to right-censored survival data [36, 38, 40, 48] in order to apply to left-censoring due to detection limits. Foreseeably, in our homoscedastic Gaussian outcome data scenario, the Gaussian Buckley-James showed better behavior than the non-parametric algorithm. However, accounting for the knowledge about the distribution seems to have had a slight influence. When the Gaussian assumption is violated, non-parametric imputation using Kaplan Meier is perhaps the best option.

We provide a publicly available R code to compute the methods introduced in this work (https://github.com/psBiostat/left-censored-Lasso). It would be interesting to compare the Lasso-regularized Buckley-James least squares method to Lasso-regularized censored LAD method. The Lasso extension of censored LAD has been proposed in different works [41, 42, 56–62]. However, to our knowledge, there is no publicly available implementation, and no simple implementation relying on existing packages seem straightforward. Moreover, several works have shown the superiority of maximum likelihood estimation in low-dimensional settings when the Gaussian assumption is valid [18, 20–22]. Nevertheless, optimization strategies for complex likelihood functions (such as that in Eq. 7) including penalties that are not smooth are not obvious.

To illustrate the application of the methods on real data, we consider a data set from the Standardization and Clinical Relevance of HIV Drug Resistance Testing Project for the Forum for Collaborative HIV Research. The data set used to illustrate the initial data set is characterized by a sample size-to-predictors ratio of around 1. There is no gold standard to measure and compare predictive performance of the different methods when using censored outcome data. All patients were being treated with abacavir, an NRTI, so we expected our methods to select a high number of HIV genotypic mutations known to contribute to abacavir and NRTI resistance. Furthermore, a high number of patients were on PI- and/or NNRTI-containing regimens, and a selection of several HIV genotypic mutations reported to be probably associated with resistance to any of these molecules was also expected [73, 74]. In that sense, the Gaussian and non-parametric Buckley-James methods showed more coherent results with the literature compared to simple imputation.

Otherwise, the data presented a moderate censoring rate of 26%, which is a realistic magnitude [18, 50] in studies measuring HIV viral load. However, high or even severe censoring rates were found in older studies with a high limit of detection (LOD) or particular populations with a low treatment failure rate [18, 51, 52]. Furthermore, left-censoring due to the lower detection limit of an assay is a problem in many fields such as biology, immunology, chemistry, and the environmental sciences in which high censoring rates may be frequent. Our simulation study shows that the difference in performance between Lasso-regularized Buckley-James methods and Lasso-regularized simple imputation methods increased with the censoring rate.

In our simulations and real application, the detection threshold was the same for all subjects. The detection threshold may vary among subjects, for example, in multicentric studies. Our R code also supports multiple lower limits of quantification. However, the findings should be interpreted with caution: differences in technological equipment could be a confounding factor that might help explain the differences in patient response to HIV treatment (in addition to HIV mutations). Adjusting or stratifying for the hospital would then be necessary.

In this study we focused on the prediction performance of Lasso-regularized methods. In clinical applications, even when prediction accuracy is the main objective, researchers aim to identify which predictors are more strongly associated with outcome. Our proposal could be easily extended or adapted to support other Lasso-type penalties. When the primary goal is to infer the set of truly relevant variables, the adaptive Lasso and the bootstrap-enhanced Lasso could thus be considered.
